# A Critical Four‐Hour Therapeutic Window Predicts Delayed Encephalopathy Risk After Carbon Monoxide Poisoning: A Multicenter Retrospective Cohort Study

**DOI:** 10.1002/cns.70837

**Published:** 2026-03-20

**Authors:** Shaokun Wang, Yanxia Gao, Jie Ran, Yan Zhang, Yanling Chen, Hongyi Yan, Li Pang

**Affiliations:** ^1^ Department of Emergency The First Hospital of Jilin University Changchun Jilin People's Republic of China; ^2^ Department of Emergency Medicine The First Affiliated Hospital of Zhengzhou University, Medical Key Laboratory of Poisoning Diseases of Henan Province Zhengzhou Henan People's Republic of China; ^3^ Department of Pediatrics Children's Medical Center, the First Hospital of Jilin, Lequn Branch Changchun Jilin People's Republic of China

**Keywords:** carbon monoxide poisoning, cohort study, delayed encephalopathy, machine learning, risk stratification, temporal factors

## Abstract

**Aims:**

The therapeutic window for preventing delayed encephalopathy after carbon monoxide poisoning (DEACMP) remains unclear. We aimed to define this temporal risk relationship and establish an intervention threshold using machine learning.

**Methods:**

In this multicenter retrospective cohort study (*n* = 1755), a gradient boosting model for predicting DEACMP was developed (*n* = 1654) and externally validated (*n* = 101). Performance was assessed using the area under the receiver operating characteristic curve (AUC) and interpreted using Shapley Additive exPlanations (SHAP).

**Results:**

The exposure‐to‐treatment interval was the most powerful predictor of DEACMP risk. Intervention within four hours emerged as the most critical variable influencing risk (SHAP analysis). The model demonstrated robust discrimination in the training (AUC = 0.944, 95% CI, 0.926–0.960), internal validation (AUC = 0.849, 95% CI, 0.785–0.905), and external validation (AUC = 0.872, 95% CI, 0.772–0.946) sets.

**Conclusion:**

Treatment delay is the primary modifiable risk factor for DEACMP following CO poisoning. The identified critical four‐hour therapeutic window provides the first quantitative, evidence‐based benchmark to inform clinical guidelines and optimize emergency response strategies aimed at preventing delayed neurological sequelae.

## Graphical abstract

A multicenter, machine learning study finds the exposure‐to‐treatment interval is the most powerful predictor of delayed encephalopathy (DEACMP) after carbon monoxide poisoning. This work identifies a critical four‐hour therapeutic window, providing an evidence‐based benchmark for preventing neurological sequelae.

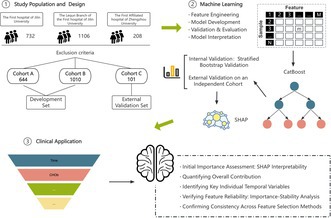

## 1 Introduction

Carbon monoxide (CO) poisoning, resulting from the incomplete combustion of carbonaceous fuels, remains a persistent and significant cause of central nervous system (CNS) injury globally. In the United States alone, unintentional CO exposure accounts for more than 400 deaths and approximately 100,000 emergency department visits annually [1] with its incidence showing a marked increase in regions such as China over the past two decades [2]. The primary pathophysiology involves the high affinity of CO for hemoglobin, which severely impairs systemic oxygen transport and can lead to profound neurological damage, myocardial injury, and death [3]. A particularly devastating neurological complication is delayed encephalopathy after acute carbon monoxide poisoning (DEACMP), which affects up to 40% of survivors [4]. This condition is characterized by a deceptive lucid interval, during which patients may initially appear to recover but later develop debilitating neurological deficits, including cognitive decline, motor dysfunction, and extrapyramidal symptoms [5].

Given the limited efficacy of therapies once DEACMP manifests, prevention through early risk stratification and timely intervention is crucial for mitigating long‐term neurological harm [6]. However, the insidious symptom‐free period frequently delays presentation, causing many patients to miss the optimal therapeutic window [7]. While prompt intervention is widely accepted as beneficial, the precise temporal threshold within which treatment most effectively prevents DEACMP remains undefined. Consequently, current clinical guidelines lack specific evidence‐based recommendations regarding the optimal time‐to‐treatment window, hindering effective early risk assessment and therapeutic prioritization.

This knowledge gap highlights the urgent need to quantify the relationship between intervention timing and DEACMP risk, directly informing neuroprotective therapeutic strategies [8, 9]. Advanced analytical methods such as machine learning offer the potential to unravel complex, nonlinear associations within clinical data and identify critical temporal factors influencing neurological outcomes. Therefore, this study aimed to leverage machine learning techniques within a large multicenter cohort to precisely quantify the association between the exposure‐to‐treatment interval and DEACMP incidence. We hypothesized that this interval is a primary determinant of DEACMP risk and that establishing a quantitative threshold could provide an actionable, evidence‐based benchmark for clinical practice and public health strategies aimed at preventing CNS sequelae.

## 2 Methods

### 2.1 Study Design and Population

This multicenter retrospective cohort study used data from three hospitals in northern China. This study adhered to the Strengthening of the Reporting of Observational Studies in Epidemiology (STROBE) guidelines (Table S1). Patients who presented to the emergency department with CO poisoning between January 2010 and December 2023 (cohorts A and B) or between January 2020 and December 2023 (cohort C) were included in the study.

Inclusion criteria: (1) carboxyhemoglobin (COHb) levels ≥ 5% (≥ 10% in smokers) at the time of medical treatment and/or documented history of CO exposure; and (2) complete follow‐up data available for at least 6 weeks to assess DEACMP, as this condition typically manifests within six weeks of initial exposure [4]. Exclusion criteria: (1) mixed poisoning involving concurrent drug overdose or alcohol intoxication; (2) altered consciousness attributable to acute cerebrovascular disease; (3) pre‐existing cerebrovascular disease that could confound DEACMP assessment; and (4) incomplete data for essential predictor variables.

**FIGURE 1:**
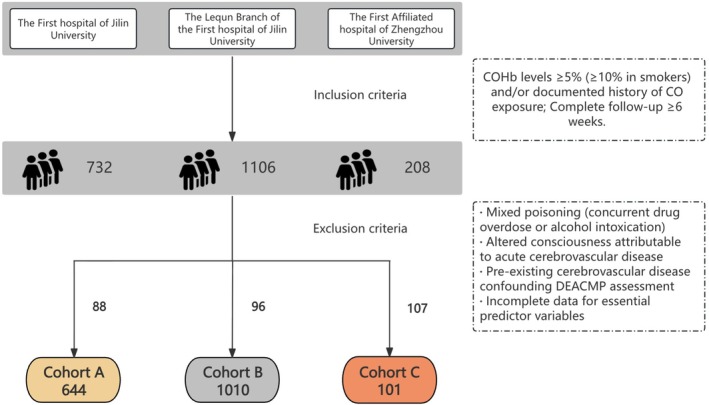
Flowchart of participant selection and study design.

The final cohort comprised a total of 1755 patients. To rigorously evaluate model generalizability [10, 11] and address potential distribution shifts between clinical settings [12, 13], patients were allocated to a development cohort (*n* = 1654 from two centers) and an independent external validation cohort (*n* = 101 from a third center), as depicted in the study flowchart (Figure 1). The development cohort was further divided into the training and internal validation sets. The overall study size was determined by consecutive enrollment of all eligible patients who met the criteria during the specified periods.

### 2.2 Ethical Considerations

The study protocol was approved by the Human Subject Ethics Committee of the First Hospital of Jilin University (Ethics Code: 21 K042‐001). Given the retrospective nature of the study and the use of routinely collected clinical data, the requirement for individual informed consent was waived by the Institutional Review Board. This study was conducted in accordance with the principles of the Declaration of Helsinki.

### 2.3 Data Collection and Outcome Definition

Data were retrospectively collected from the electronic medical records. The primary exposure variable was the interval between CO exposure and initiation of medical treatment, recorded in hours and based on patient or witness reports. Carboxyhemoglobin (COHb) levels on admission were measured using co‐oximetry. Owing to the rapid decline in COHb post‐exposure, the Glasgow Coma Scale (GCS) score at admission served as a surrogate indicator of initial poisoning severity. Additional baseline covariates included demographics (age and sex), comorbidities (hypertension, diabetes mellitus, coronary heart disease, prior cerebrovascular disease, smoking status, and alcohol consumption), vital signs, and laboratory results (blood samples drawn upon admission). Information regarding treatments, including hyperbaric oxygen therapy, was also documented.

The primary outcome was the occurrence of DEACMP, defined according to established clinical criteria, within six weeks of initial CO exposure. Patients were followed up through a medical record review.

### 2.4 Statistical Analysis and Machine Learning Model

Descriptive statistics were computed for the baseline characteristics. Continuous variables are presented as median (interquartile range [IQR]) and were compared using the Mann–Whitney U test. Categorical variables are presented as frequencies (percentages) and were compared using Fisher's exact test. The domain shift between the development and external validation cohorts was evaluated by comparing the DEACMP prevalence [14].

A gradient‐boosting machine learning approach using the CatBoost algorithm was employed to predict DEACMP, chosen for its proficiency with clinical datasets and intrinsic handling of missing data, which were minimal in this cohort (<1%) [15]. The modeling workflow involved several steps.

- Model Construction: A stratified domain‐adaptive allocation strategy was used for the training and internal validation sets. Class imbalance was addressed using class‐balanced weighting (Table S1).
- Feature Engineering and Selection: Advanced feature engineering was systematically applied to create temporal, mathematical, and interactive features, as follows: a detailed list of engineered features is provided in Tables S2–S9. Combined feature selection strategies, including statistical F‐tests and mutual information approaches, were used (Table S2).
- Model Validation: Internal validation was performed using a stratified bootstrap framework (500–1000 iterations) to assess the model stability. External validation was performed on a completely independent cohort without retention or parameter tuning, following the established guidelines for clinical prediction models [16] (Table S3).
- Performance Assessment and Interpretation: Model discrimination was evaluated using the area under the receiver operating characteristic curve (AUC) as the primary metric. Differences in AUCs between the models were assessed using DeLong's test. Model predictions were made interpretable using SHAP (SHapley Additive exPlanations) values applied to representative patient samples, [17, 18] and an importance‐stability analysis was conducted to identify reliable features (Table S4).
- To assess the clinical utility of the model beyond standard performance metrics, decision curve analysis (DCA) was performed for both the internal and external validation sets. DCA calculates the net clinical benefit of the prediction model across a range of threshold probabilities and compares it to the default strategies of treating all or no patients.

All analyses were performed using Python 3.9 with established clinical machine‐learning libraries (scikit‐learn, CatBoost, and SHAP). All P‐values were two‐sided and reported as exact values where appropriate.

## 3 Results

### 3.1 Characteristics of Participants

The proportion of missing data among the baseline covariates was minimal (< 1%) and was inherently handled by the CatBoost algorithm. After applying the eligibility criteria, 1755 patients were enrolled in this study, with 1654 patients allocated to the development cohort (1481 non‐DEACMP and 173 DEACMP) and 101 patients assigned to the external validation cohort (59 non‐DEACMP and 42 DEACMP). The development cohort was subsequently divided into the training (*n* = 1270) and internal validation (*n* = 384) subsets.

In the development cohort, patients in the DEACMP group were older than those in the non‐DEACMP group (median age, 56.0 vs. 54.0 years; median difference, 2.0 years; 95% CI, −0.7 to 4.7; *p* = 0.009), and had a lower proportion of males (40.5% vs. 49.0%; difference, −8.5%; 95% CI, −16.2% to −0.7%; *p* = 0.042). Patients with DEACMP also had longer exposure‐to‐presentation intervals (9.0 vs. 7.0 h, *p < 0.001)* and a higher prevalence of diabetes mellitus (19.1% vs. 4.0%; difference, 15.1%; 95% CI, 9.2% to 21.0%; *p < 0.001)* and coronary heart disease (15.0% vs. 4.1%; difference, 10.9%; 95% CI, 5.5% to 16.3%; *p < 0.001)*. Conversely, they presented with lower admission COHb levels (median, 12.6% vs. 18.6%; median difference, −6.0%; 95% CI, −8.3% to −3.7%; *p < 0.001)*. Intergroup differences were also observed in the external validation cohort (Table 1).

**TABLE 1:** Baseline demographic and clinical characteristics of patients with and without delayed encephalopathy after carbon monoxide poisoning (DEACMP) in the development and external validation cohorts.

Characteristic	Development set (cohort A + B)				External validation set (cohort C)			
	Non‐DEACMP (*n* = 1481)	DEACMP (*n* = 173)	Difference (95% CI)	*p*	Non‐DEACMP (*n* = 59)	DEACMP (*n* = 42)	Difference (95% CI)	*p*
**Demographic characteristics**								
Sex (male), *n* (%)	725 (49.0)	70 (40.5)	−8.5 (−16.2, −0.7)	0.042	31 (52.5)	16 (38.1)	−14.4 (−33.9, 5.0)	0.218
Age (years), M (Q1, Q3)	54.0 (40.0, 65.0)	56.0 (49.0, 66.0)	2.0 (−0.7, 4.7)	0.009	44.0 (24.5, 65.5)	61.0 (53.0, 68.8)	17.0 (9.0, 25.0)	< 0.001
**Clinical characteristics**								
Time from exposure (hours), M (Q1, Q3)	7.0 (4.0, 12.0)	9.0 (5.0, 24.0)	2.0 (−35.6, 39.6)	< 0.001	24.0 (7.0, 96.0)	612.0 (342.0, 720.0)	588.0 (177.4, 998.6)	< 0.001
GCS score, M (Q1, Q3)	15.0 (15.0, 15.0)	15.0 (15.0, 15.0)	0.0 (−0.6, 0.6)	0.753	13.0 (6.0, 15.0)	12.0 (10.0, 13.0)	−1.0 (−2.5, 0.5)	0.062
**Comorbidities, *n* (%)**								
Diabetes mellitus	59 (4.0)	33 (19.1)	15.1 (9.2, 21.0)	< 0.001	4 (6.8)	6 (14.3)	7.5 (−4.9, 19.9)	0.364
Hypertension	180 (12.2)	31 (17.9)	5.8 (−0.2, 11.7)	0.042	14 (23.7)	15 (35.7)	12.0 (−6.1, 30.1)	0.276
Coronary heart disease	61 (4.1)	26 (15.0)	10.9 (5.5, 16.3)	< 0.001	3 (5.1)	5 (11.9)	6.8 (−4.5, 18.1)	0.380
Cerebrovascular disease	68 (4.6)	15 (8.7)	4.1 (−0.2, 8.4)	0.032	6 (10.2)	9 (21.4)	11.3 (−3.4, 25.9)	0.199
Smoking	242 (16.3)	42 (24.3)	7.9 (1.3, 14.6)	0.012	2 (3.4)	8 (19.0)	15.7 (2.9, 28.4)	0.024
Drinking	155 (10.5)	21 (12.1)	1.7 (−3.4, 6.8)	0.586	2 (3.4)	2 (4.8)	1.4 (−6.6, 9.3)	1.000
**Vital signs, M (Q1, Q3)**								
Height (cm)	165.0 (162.0, 168.9)	165.0 (162.0, 167.6)	0.0 (−1.6, 1.6)	0.542	165.6 (164.6, 168.3)	164.4 (163.2, 167.6)	−1.2 (−2.6, 0.2)	0.060
Weight (kg)	63.3 (60.0, 66.0)	63.0 (60.0, 64.2)	−0.3 (−1.7, 1.1)	0.066	63.1 (59.2, 66.2)	63.6 (62.3, 68.2)	0.5 (−1.2, 2.2)	0.015
BMI (kg/m^2^)	23.1 (22.6, 23.9)	23.1 (23.1, 23.2)	0.0 (−0.4, 0.4)	0.621	22.5 (22.1, 23.1)	23.4 (22.3, 24.4)	0.9 (0.4, 1.4)	0.004
SBP (mmHg)	125.0 (122.0, 128.0)	125.0 (125.0, 125.0)	0.0 (−4.8, 4.8)	0.491	123.0 (120.0, 125.5)	124.0 (123.0, 127.0)	1.0 (−0.6, 2.6)	0.004
DBP (mmHg)	75.0 (74.0, 78.0)	75.0 (75.0, 75.0)	0.0 (−1.1, 1.1)	0.288	75.0 (74.0, 77.0)	75.0 (74.2, 76.0)	0.0 (−1.0, 1.0)	0.341
**Laboratory tests, M (Q1, Q3)**								
pH	7.4 (7.4, 7.4)	7.4 (7.4, 7.5)	0.0 (−0.1, 0.1)	0.112	7.4 (7.4, 7.4)	7.4 (7.4, 7.5)	0.0 (0.0, 0.1)	< 0.001
PCO2 (mmHg)	36.0 (35.0, 37.0)	36.0 (36.0, 36.0)	0.0 (−1.9, 1.9)	0.251	36.0 (33.0, 40.0)	36.0 (35.0, 39.0)	0.0 (−2.0, 2.0)	0.688
pO2 (mmHg)	86.0 (81.0, 90.0)	86.0 (82.0, 86.0)	0.0 (−3.9, 3.9)	0.063	97.0 (83.5, 142.5)	95.5 (87.0, 108.0)	−1.5 (−17.9, 14.9)	0.436
Sodium (mmol/L)	138.0 (136.4, 139.5)	138.0 (136.1, 140.0)	0.0 (−1.9, 1.9)	0.349	137.6 (137.0, 138.4)	137.6 (136.7, 138.4)	−0.0 (−0.5, 0.4)	0.384
Potassium (mmol/L)	3.7 (3.4, 3.9)	3.7 (3.4, 3.9)	0.0 (−0.4, 0.4)	0.867	3.6 (3.5, 3.7)	3.8 (3.7, 3.8)	0.2 (0.2, 0.2)	< 0.001
Calcium (mmol/L)	1.1 (1.1, 1.1)	1.1 (1.1, 1.1)	0.0 (−175.9, 175.9)	0.927	1.1 (1.1, 1.1)	1.1 (1.1, 1.1)	0.0 (0.0, 0.1)	< 0.001
Glucose (mmol/L)	6.9 (6.1, 8.4)	7.3 (5.9, 8.8)	0.4 (−0.1, 0.9)	0.352	6.5 (5.8, 7.3)	7.3 (6.8, 7.9)	0.8 (0.5, 1.2)	< 0.001
Lactate (mmol/L)	2.0 (1.4, 3.3)	1.9 (1.1, 3.9)	−0.1 (−0.5, 0.3)	0.054	1.3 (0.8, 1.9)	1.2 (1.1, 1.4)	−0.1 (−0.5, 0.3)	0.945
COHb (%)	18.6 (10.0, 28.8)	12.6 (4.3, 19.5)	−6.0 (−8.3, −3.7)	< 0.001	6.3 (1.1, 18.3)	2.2 (1.3, 3.8)	−4.0 (−7.6, −0.5)	0.019
HCO3‐ (mmol/L)	23.0 (20.8, 24.6)	22.8 (19.0, 24.8)	−0.2 (−0.8, 0.4)	0.352	22.8 (21.4, 24.4)	25.3 (23.4, 25.8)	2.5 (1.3, 3.7)	< 0.001
WBC (×10^9^/L)	9.3 (8.3, 10.4)	9.3 (9.0, 9.3)	0.0 (−3.9, 3.9)	0.290	9.8 (6.9, 13.0)	6.9 (5.2, 9.6)	−2.9 (−5.1, −0.7)	0.001
Hemoglobin (g/L)	139.3 (135.0, 145.0)	139.3 (137.3, 139.3)	0.0 (−2.5, 2.5)	0.136	138.0 (133.8, 143.1)	139.3 (129.9, 140.4)	1.3 (−2.2, 4.8)	0.574
Platelets (×10^9^/L)	222.0 (208.7, 242.0)	222.0 (222.0, 225.0)	0.0 (−8.0, 8.0)	0.592	205.0 (158.0, 231.0)	193.0 (158.0, 260.0)	−12.0 (−39.4, 15.4)	0.880
BUN (mmol/L)	5.3 (4.8, 5.8)	5.3 (5.3, 5.3)	0.0 (−0.4, 0.4)	0.505	5.8 (4.5, 7.2)	5.1 (3.9, 6.4)	−0.7 (−8.7, 7.2)	0.215
Creatinine (μmol/L)	63.0 (59.0, 67.5)	63.0 (63.0, 63.8)	0.0 (−3.4, 3.4)	0.511	61.0 (48.5, 74.0)	55.0 (48.4, 63.8)	−6.0 (−27.0, 15.0)	0.228
Albumin (g/L)	41.3 (40.2, 42.2)	41.3 (40.0, 41.3)	0.0 (−0.6, 0.6)	0.002	41.9 (41.0, 43.1)	41.3 (40.3, 43.5)	−0.6 (−1.8, 0.6)	0.309
**Cardiac biomarkers, M (Q1, Q3)**								
Myoglobin (ng/mL)	29.2 (13.0, 45.8)	23.5 (5.0, 40.6)	−5.7 (−89.2, 77.8)	0.059	340.9 (178.4, 503.1)	502.9 (177.5, 502.9)	162.0 (−11.6, 335.7)	0.651
Troponin (ng/mL)	0.1 (0.0, 0.2)	0.1 (0.1, 0.1)	0.0 (−0.5, 0.5)	0.679	0.3 (0.1, 0.4)	0.2 (0.0, 0.3)	−0.1 (−0.2, 0.0)	0.160
BNP (pg/mL)	231.0 (76.0, 926.0)	318.0 (80.0, 1623.0)	87.0 (−484.5, 658.5)	0.168	727.5 (463.0, 886.0)	345.0 (252.5, 562.5)	−382.5 (−832.6, 67.6)	0.489
EF (%)	62.0 (61.0, 63.0)	62.0 (58.0, 63.0)	0.0 (−1.1, 1.1)	0.849	55.0 (55.0, 61.0)	61.0 (45.0, 61.0)	6.0 (−3.7, 15.7)	1.000
**Treatment, *n* (%)**								
Hyperbaric oxygen therapy	1419 (95.8)	169 (97.7)	1.9 (−0.6, 4.3)	0.324	45 (76.3)	10 (23.8)	−52.5 (−69.3, −35.6)	< 0.001

*Note:* Development Set (Cohort A + B): Total *n* = 1654, Non‐DEACMP *n* = 1481, DEACMP *n* = 173. External Validation Set (Cohort C): Total *n* = 101, Non‐DEACMP *n* = 59, DEACMP *n* = 42. Data are presented as median (interquartile range) for continuous variables and *n* (%) for categorical variables. The Mann–Whitney *U* test was used for continuous variables and Fisher's exact test for categorical variables. *p* values < 0.05 were considered statistically significant.

Abbreviations: BMI, body mass index; BNP, B‐type natriuretic peptide; BUN, blood urea nitrogen; CI, confidence interval; COHb, carboxyhemoglobin; DBP, diastolic blood pressure; DEACMP, delayed encephalopathy after carbon Mmnoxide poisoning; EF, ejection fraction; GCS, glasgow coma scale; M, median; Q1, first quartile; Q3, third quartile; SBP, systolic blood pressure; WBC, white blood cell.

### 3.2 Model Performance and Domain Adaptation

**FIGURE 2:**
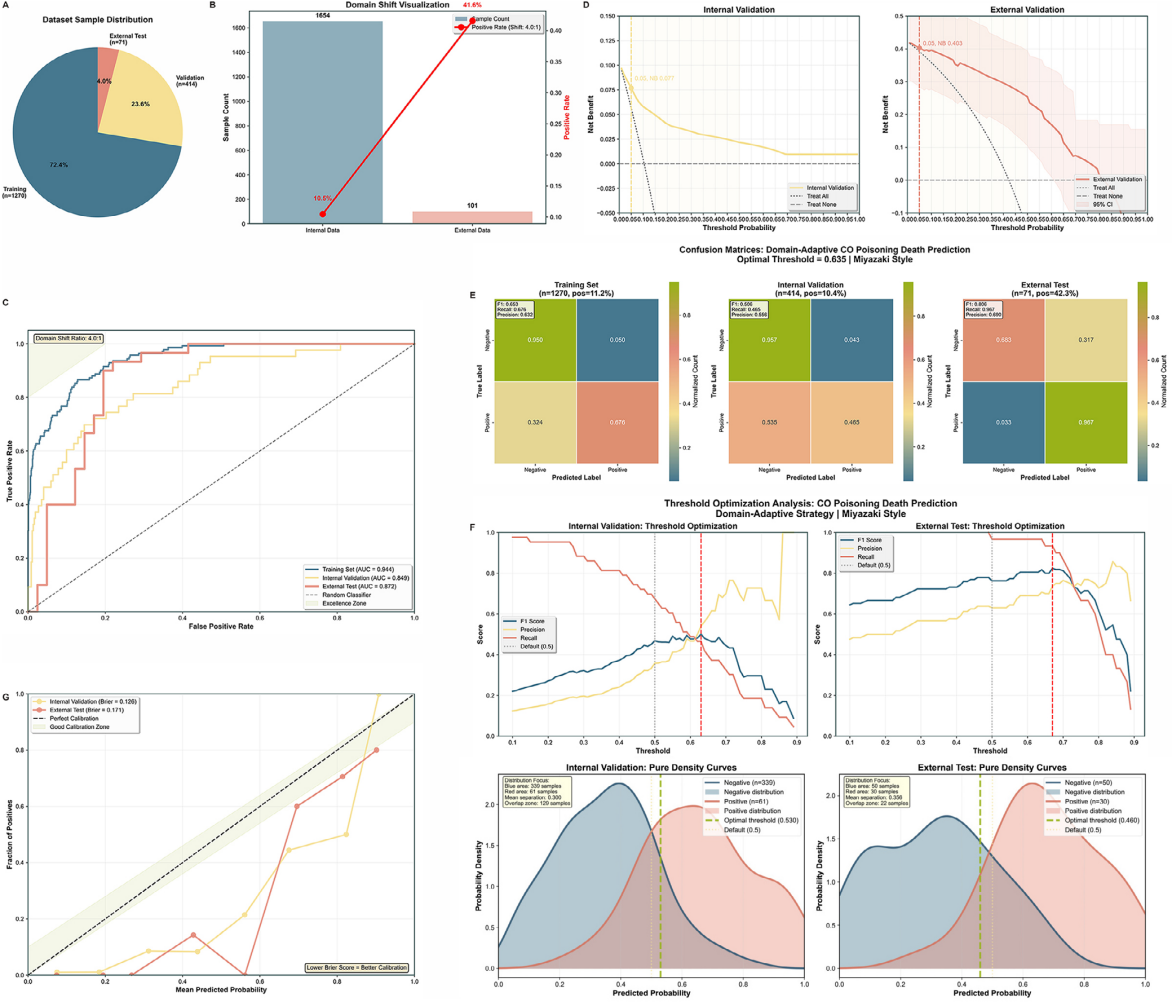
Model performance, domain adaptation, and validation. (A) Pie chart illustrating the sample distribution between the development set (training and internal validation) and the external test set. (B) Bar chart visualizing the significant domain shift, showing the DEACMP (delayed encephalopathy after carbon monoxide poisoning) positive rate in the development cohort (10.5%; blue bar) versus the external validation cohort (41.6%; orange bar). (C) Receiver Operating Characteristic (ROC) curves for the training (AUC = 0.944; black line), internal validation (AUC = 0.849; orange line), and external validation (AUC = 0.872; red line) datasets, demonstrating the model's robust discriminative ability. (D) Decision Curve Analysis (DCA) for the internal (left) and external (right) validation cohorts. The y‐axis measures the net benefit. The yellow (internal) and red (external) lines indicate the model's net benefit, which consistently exceeds the reference strategies of “Treat All” (gray line) and “Treat None” (black line) across a wide range of threshold probabilities, demonstrating clinical utility. (E) Normalized confusion matrices detailing the model's classification accuracy. Notably, the model achieved an exceptionally high sensitivity of 0.967 on the external validation set, indicating its strong capability in identifying high‐risk DEACMP patients. (F) Threshold optimization curves for internal and external validation, plotting F1‐score (black line), precision (orange line), and recall (yellow line) against prediction thresholds to identify optimal operating points, which were 0.530 for internal and 0.460 for external validation. (G) Calibration plots (top panels) and probability density curves (bottom panels). The calibration plots assess the model's probability accuracy, yielding Brier scores of 0.126 for internal (orange line) and 0.171 for external (red line) validation. The density curves illustrate the separation of predicted probabilities for negative (blue distribution) and positive (red distribution) cases.

A significant 4.0‐fold difference in DEACMP incidence was observed between the external validation (41.6%) and development cohorts (10.5%), highlighting a substantial domain shift (Figure 2A,B). Critically, the domain‐adaptive CatBoost model demonstrated high robustness to this distributional heterogeneity, maintaining a strong predictive performance across all sets. As depicted in Figure 2C, the model achieved an AUC of 0.944 (95% CI, 0.926 to 0.960) for the training set, 0.849 (95% CI, 0.785 to 0.905) for internal validation, and 0.872 (95% CI, 0.772 to 0.946) for the external validation set.

The DCA demonstrated substantial clinical value of the model (Figure 2D). In both internal and external validation sets, the model's net benefit curve (solid lines) consistently remained above the reference lines for “treat‐all” and “treat‐none” strategies across a broad range of threshold probabilities (approximately 0.05 to 0.80). This indicates that applying the model to guide therapeutic interventions yields a positive net benefit relative to the default protocols regardless of the clinician's preference for the risk threshold.

The detailed performance metrics are presented in Figure 2E. In the training set, the F1‐score was 0.653 (95% CI, 0.627 to 0.679). The internal validation set yielded an F1‐score of 0.506 (95% CI, 0.458 to 0.554). For the external validation set, the model achieved a sensitivity of 0.967 (95% CI, 0.925 to 1.000) and an F1‐score of 0.806 (95% CI, 0.713 to 0.898). The optimal performance thresholds were 0.530 for internal validation and 0.460 for external validation (Figure 2F). Model calibration was evaluated using Brier scores of 0.126 and 0.171 for internal and external validation, respectively (Figure 2G).

### 3.3 Temporal Factors in DEACMP Prediction

**FIGURE 3:**
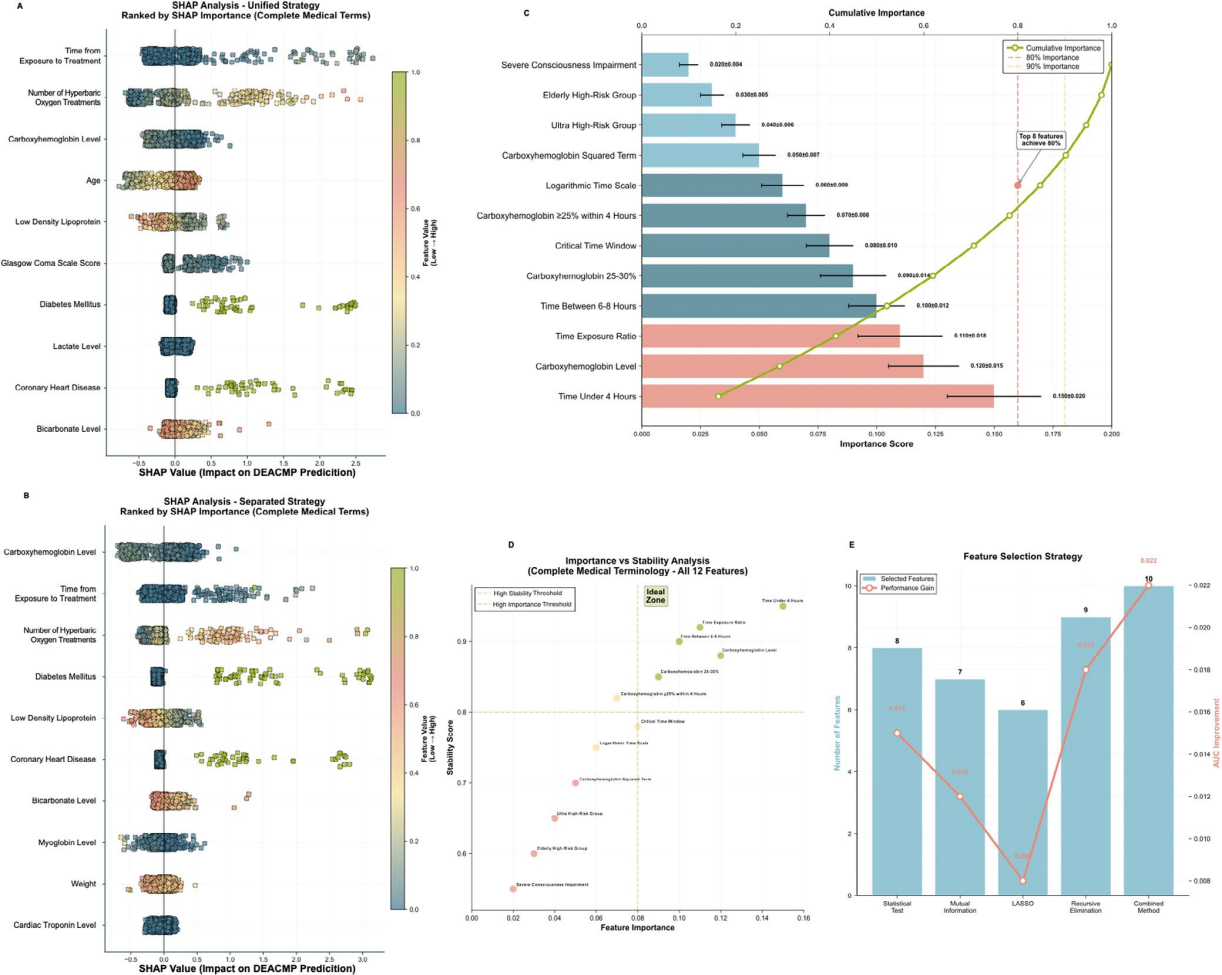
Model interpretability and feature importance analysis. (A) SHAP (SHapley Additive exPlanations) summary plot for the unified analysis strategy, ranking the top 10 predictors by their mean absolute SHAP value. Each point represents a patient; its position on the x‐axis indicates the feature's impact on the DEACMP prediction, and its color represents the feature's value (red for high, blue for low). In this unified view, Time from Exposure to Treatment is identified as the most influential predictor. (B) SHAP summary plot for the separated analysis strategy, demonstrating how predictor importance shifts in a dataset‐specific context, with “Carboxyhemoglobin Level” emerging as the dominant feature. (C) Bar chart of the top individual engineered features ranked by importance score, with an overlaid curve showing their cumulative contribution (yellow line). The analysis highlights that the top eight features account for 80% of the model's total predictive capacity, with Time Under Four Hours being the single most important variable. (D) Importance versus stability analysis plot, which identifies features located in the “Ideal Zone” (importance > 0.08, stability > 0.8). Three temporal variables were identified as being both highly important and stable across bootstrap iterations. (E) Feature selection analysis showing the number of features selected by four different methods and a combined approach (blue bars), along with the corresponding improvement in AUC performance (red line). The combined method using 10 features yielded the optimal performance gain. AUC, area under the receiver operating characteristic curve; DEACMP, delayed encephalopathy after carbon monoxide poisoning; SHAP, Shapley Additive exPlanations.

Model interpretability was evaluated using SHAP analysis (Figure 3A,B). The unified strategy identified time to presentation as the primary predictor (mean absolute SHAP value = 0.334), followed by hyperbaric oxygen treatment frequency (0.258) and COHb levels (0.193). These findings were corroborated by CatBoost‐derived feature importance rankings (Figure S1). A comprehensive feature importance analysis was conducted, in which robust external validation performance provided a reliable foundation (Figure S2). This analysis confirmed that temporal features collectively accounted for 39.1% of the total predictive importance, representing the largest contribution among all feature categories (Figure S3).

Among individual engineered features, presentation time ≤ 4 h had the highest individual importance score (0.150 ± 0.020), followed by COHb levels (0.120 ± 0.015) and presentation time ratio (0.110 ± 0.018). Cumulative importance analysis revealed that the eight most influential features captured 80% of the total predictive capacity of the model, with five being temporally derived variables (Figure 3C). Importance‐stability analysis identified presentation time ≤ 4 h as having high importance (> 0.08) and high stability (> 0.8) across multiple model iterations (Figure 3D). Furthermore, combined feature‐selection strategies using 10 features yielded an AUC improvement of 0.022 compared to the full feature set, with temporal variables consistently selected across all methods (Figure 3E).

## 4 Discussion

In this multicenter cohort study, we developed and externally validated a machine learning model for DEACMP prediction, establishing that the temporal interval between carbon monoxide exposure and medical intervention is the most powerful predictor. Our principal finding is the quantitative identification of a critical therapeutic window, with intervention within the first four hours being paramount for reducing risk.

By employing a novel domain‐adaptive framework, our model successfully navigated the substantial heterogeneity inherent in real‐world clinical data, demonstrating robust predictive accuracy and generalization in an independent validation cohort. This approach translates complex clinical data into an actionable tool, offering clinicians crucial prognostic insights during the early hours of patient care.

Our central finding, the paramount importance of the exposure‐to‐treatment interval, is strongly corroborated by existing clinical evidence. A key study by Lee et al. demonstrated that patients receiving hyperbaric oxygen therapy within six hours of CO exposure had substantially better six‐month neurocognitive outcomes than those treated later; their work further established a dose–response relationship where treatment delay was directly associated with an increased incidence of poor prognoses [6]. This aligns with the findings from the landmark randomized controlled trial by Weaver et al., which established the efficacy of hyperbaric oxygen based on a cohort where the majority of patients were treated within this critical six‐hour window [19]. This evidence is further reinforced by foundational studies from Thom et al. and Ducassé et al., which similarly identified prompt intervention as essential [20, 21]. From a mechanistic standpoint, the urgency for rapid treatment is grounded in the time‐dependent nature of CO‐induced cellular injury. Research by Brvar et al. has shown that the neuroprotective effect of hyperbaric oxygen against neuronal apoptosis is most potent within the initial hours post‐exposure [22]. Therefore, the high predictive weight our model assigns to temporal factors, particularly within a four‐hour window, is not merely a statistical correlation but a reflection of the fundamental, time‐sensitive pathophysiology of CO‐induced brain injury.

We rigorously evaluated the reliability of this four‐hour threshold to ensure that it was not a statistical artifact. First, from a data‐driven perspective, our importance‐stability analysis (Figure 3D) demonstrated that treatment within four hours was consistently the most stable predictor across 1000 bootstrap iterations (stability score > 0.8), significantly outperforming other temporal cut‐offs. Second, the robustness of this window was validated using an independent external cohort. Despite the substantial domain shift in disease prevalence and geographic location, the four‐hour window remained the dominant risk factor, confirming its generalizability beyond the training site. Third, compared to the traditionally cited six‐hour window, our identified four‐hour threshold represents a more precise inflection point for neuroprotection. This narrower window is consistent with that observed in animal models, suggesting that the cascade of mitochondrial oxidative stress and leukocyte sequestration becomes increasingly irreversible after the first few hours. Thus, the four‐hour window offers a more sensitive target for emergency triage than previously established benchmarks.

Although our model emphasizes the exposure‐to‐treatment interval, its predictive power must be contextualized by the initial severity of poisoning. Notably, Lee et al. found that in the most severely poisoned patients, the benefit of early versus late treatment on neurocognitive outcomes was not demonstrated [6]. This suggests that a profound initial hypoxic–ischemic insult may be the dominant prognostic factor. This concept is supported by the complex cascade of secondary injuries, such as oxidative stress and neuroinflammation, that follow CO exposure and contribute to the final outcome [3]. Therefore, while rapid intervention is crucial, the ultimate prognosis is likely determined by an interplay between treatment timing and the severity of the initial insult.

Our findings also clarify the “COHb paradox,” where patients who developed DEACMP often presented with lower admission COHb levels. This observation likely does not reflect a lower initial poisoning severity but rather a longer delay in seeking medical care, a period during which COHb levels decline rapidly. This scenario underscores why our model correctly identified time‐to‐presentation as a more robust predictor of long‐term neurological harm than admission COHb level, which can be misleading if the presentation is delayed. Furthermore, despite a four‐fold difference in DEACMP incidence, representing a significant real‐world domain shift, the underlying logic of the model holds true. Its ability to generalize and reliably identify high‐risk patients across heterogeneous clinical settings confirms its utility as a robust clinical decision‐support tool.

Although the model demonstrated robust discrimination across cohorts, variations in performance metrics were observed. The F1‐score of the external validation set (0.806) was significantly higher than that of the internal validation set (0.506). This discrepancy was primarily improved by the significantly higher prevalence of DEACMP in the external cohort (41.6% vs. 10.5%), highlighting the impact of disease prevalence on precision‐recall metrics. Regarding the calibration, the increased Brier score in the external validation (0.171) reflects the challenge of probability estimation under such substantial domain shifts. Despite this calibration drift, the discriminative ability of the model remained robust (AUC = 0.872). To further evaluate the clinical utility beyond standard metrics, we performed a DCA (Figure 2D). The results demonstrated that our model provides a higher net benefit than “treat‐all” or “treat‐none” strategies across a wide range of threshold probabilities. This confirms that even if the absolute predicted probabilities fluctuate due to prevalence shifts, the model effectively stratifies risk and supports better clinical decision‐making.

Clinically, the selection of the decision threshold involves a critical tradeoff between sensitivity and specificity. In the external validation, a lower threshold of 0.460 was identified, yielding a sensitivity of 0.967. Although this lower threshold may result in an increased rate of false positives, we argue that this is clinically justifiable. In the management of DEACMP, a false‐negative (missed diagnosis) leads to irreversible neurological damage, whereas a false‐positive results in precautionary hyperbaric oxygen therapy or closer monitoring, which carries a much lower safety risk. Thus, prioritizing high sensitivity to minimize missed diagnoses is aligned with the therapeutic goal of neuroprotection.

This study has several strengths. Its multicenter design and large cohort size provided a robust foundation for the analysis. The use of a geographically and temporally independent external validation set allowed for a rigorous and unbiased assessment of the model's generalization, which is a critical step in clinical prediction model development. Methodologically, the application of an advanced, interpretable machine learning algorithm (CatBoost with SHAP analysis) enabled us to navigate complex, nonlinear relationships in the data while providing clinically understandable insights.

Nevertheless, the limitations inherent in this study warrant further investigation. First, regarding information bias, the retrospective design relied on self‐reported time from exposure to treatment, which is subject to recall bias, particularly in patients with CO‐induced consciousness disturbances. Although we attempted to corroborate the times with witness accounts and emergency medical records, some degree of imprecision was inevitable. However, the robustness of the four‐hour threshold is supported by our importance‐stability analysis (Figure 3D), in which this feature consistently emerged as the most stable predictor across 1000 bootstrap iterations. This suggests that the predictive signal of early intervention is sufficiently strong to overcome the potential noise inherent in retrospective time estimation. Second, selection bias is a potential concern. As this study was conducted in tertiary referral centers, our cohort likely represented a more severe spectrum of CO poisoning than the general population. Although this may affect the absolute prevalence of DEACMP, the domain‐adaptive design of our model ensured that it remained valid even when tested on an external cohort with a significantly different disease prevalence (Figure 2B), indicating that the core four‐hour therapeutic window is a generalizable biological feature rather than a selection artifact. Third, given that this hospital‐based cohort was drawn from tertiary centers in northern China, where coal‐burning remains a predominant source of CO exposure, the findings may not be fully generalizable to populations with different exposure patterns [23]. Future prospective validation in diverse populations is needed. Fourth, data on patient race and ethnicity were not systematically collected, preventing us from assessing potential disparities in exposure or outcomes. Finally, as in any observational study, the possibility of unmeasured confounding variables cannot be fully excluded.

## 5 Conclusion

In conclusion, this study provides robust quantitative evidence that the time between CO exposure and medical intervention is the most critical modifiable determinant of DEACMP risk. The identification of a crucial therapeutic window, with the first four hours post‐exposure being paramount, offers a clear evidence‐based benchmark for public health initiatives and emergency medical services. These findings provide clinicians with an actionable tool for early risk stratification, enabling the prioritization of high‐risk patients to mitigate the devastating long‐term neurological consequences of this common environmental hazard.

## Author Contributions

Conceptualization; Data curation; Formal analysis; Validation; Visualization; Writing – original draft: S.W. Jie Ran, Yan Zhang and Yanxia Gao: Data curation; Investigation: J.R., Y.Z, Y.G. Writing – original draft; Data curation: Y.C. Resources; Project administration: H.Y. Conceptualization; Methodology; Supervision; Funding acquisition; Writing – review and editing: L.P.

## Funding

This study was supported by the National Natural Science Foundation of China (Grant number 82372211).

## Conflicts of Interest

The authors declare no conflicts of interest.

## Supporting information


**Table S1:** STROBE checklist for observational studies.
**Table S2–S9:** Detailed list of all engineered temporal, mathematical, and interactive features.
**Figures S1–S3:** Supplementary Additional data on feature importance rankings, external validation performance, and predictive importance by feature category.
Supplementary Methods S1–S4: Technical details regarding class‐balanced weighting, feature selection strategies, external validation protocols, and importance‐stability analysis.

## Data Availability

The de‐identified patient data and the analysis code that support the findings of this study are not publicly available due to patient privacy regulations, but may be obtained from the corresponding author upon reasonable request and with the necessary ethics committee approval.
