# Supplementary material for: A Critical Four‐Hour Therapeutic Window Predicts Delayed Encephalopathy Risk After Carbon Monoxide Poisoning: A Multicenter Retrospective Cohort Study
Source: CNS Neurosci Ther. 2026 Mar 20;32(3):e70837. doi: 10.1002/cns.70837 (PMC13093570; doi:10.1002/cns.70837)
Supplement: Supplementary file 1 — Table S1: STROBE checklist for observational studies. Table S2–S9: Detailed list of all engineered temporal, mathematical, and interactive features. Figures S1–S3: Supplementary Additional data on feature importance rankings, external validation performance, and predictive importance by feature category. Supplementary Methods S1–S4: Technical details regarding class‐balanced weighting, feature selection strategies, external validation protocols, and importance‐stability analysis. [file CNS-32-e70837-s001.docx]

**Table S1. STROBE Statement—Checklist of items that should be included in reports of *cohort studies***

|  | Item No | Recommendation | Page No |
| --- | --- | --- | --- |
| **Title and abstract** | 1 | (*a*) Indicate the study’s design with a commonly used term in the title or the abstract | 1-2 |
|  |  | (*b*) Provide in the abstract an informative and balanced summary of what was done and what was found | 1-2 |
| Introduction | | | |
| Background/rationale | 2 | Explain the scientific background and rationale for the investigation being reported | 2-3 |
| Objectives | 3 | State specific objectives, including any prespecified hypotheses | 3-4 |
| Methods | | | |
| Study design | 4 | Present key elements of study design early in the paper | 4-5 |
| Setting | 5 | Describe the setting, locations, and relevant dates, including periods of recruitment, exposure, follow-up, and data collection | 4-6 |
| Participants | 6 | (*a*) Give the eligibility criteria, and the sources and methods of selection of participants. Describe methods of follow-up | 4 |
|  |  | (*b*) For matched studies, give matching criteria and number of exposed and unexposed | 4-5 |
| Variables | 7 | Clearly define all outcomes, exposures, predictors, potential confounders, and effect modifiers. Give diagnostic criteria, if applicable | 5-6 |
| Data sources/ measurement | 8* | For each variable of interest, give sources of data and details of methods of assessment (measurement). Describe comparability of assessment methods if there is more than one group | 6 |
| Bias | 9 | Describe any efforts to address potential sources of bias | 11 |
| Study size | 10 | Explain how the study size was arrived at | 4 |
| Quantitative variables | 11 | Explain how quantitative variables were handled in the analyses. If applicable, describe which groupings were chosen and why | 10 |
| Statistical methods | 12 | (*a*) Describe all statistical methods, including those used to control for confounding | 6-7 |
|  |  | (*b*) Describe any methods used to examine subgroups and interactions |  |
|  |  | (*c*) Explain how missing data were addressed |  |
|  |  | (*d*) If applicable, explain how loss to follow-up was addressed |  |
|  |  | (*e*) Describe any sensitivity analyses |  |
| Results | | |  |
| Participants | 13* | (a) Report numbers of individuals at each stage of study—eg numbers potentially eligible, examined for eligibility, confirmed eligible, included in the study, completing follow-up, and analysed | 7-8 |
|  |  | (b) Give reasons for non-participation at each stage |  |
|  |  | (c) Consider use of a flow diagram |  |
| Descriptive data | 14* | (a) Give characteristics of study participants (eg demographic, clinical, social) and information on exposures and potential confounders | 7-8 |
|  |  | (b) Indicate number of participants with missing data for each variable of interest |  |
|  |  | (c) Summarise follow-up time (eg, average and total amount) |  |
| Outcome data | 15* | Report numbers of outcome events or summary measures over time | 7-8 |

| Main results | 16 | (*a*) Give unadjusted estimates and, if applicable, confounder-adjusted estimates and their precision (eg, 95% confidence interval). Make clear which confounders were adjusted for and why they were included | 8-10 |
| --- | --- | --- | --- |
|  |  | (*b*) Report category boundaries when continuous variables were categorized |  |
|  |  | (*c*) If relevant, consider translating estimates of relative risk into absolute risk for a meaningful time period |  |
| Other analyses | 17 | Report other analyses done—eg analyses of subgroups and interactions, and sensitivity analyses | 10 |
| Discussion | | | |
| Key results | 18 | Summarise key results with reference to study objectives | 10 |
| Limitations | 19 | Discuss limitations of the study, taking into account sources of potential bias or imprecision. Discuss both direction and magnitude of any potential bias | 13-14 |
| Interpretation | 20 | Give a cautious overall interpretation of results considering objectives, limitations, multiplicity of analyses, results from similar studies, and other relevant evidence | 10-13 |
| Generalisability | 21 | Discuss the generalisability (external validity) of the study results | 10-11 |
| Other information | | | |
| Funding | 22 | Give the source of funding and the role of the funders for the present study and, if applicable, for the original study on which the present article is based | 15 |

*Give information separately for exposed and unexposed groups.

**Note:** An Explanation and Elaboration article discusses each checklist item and gives methodological background and published examples of transparent reporting. The STROBE checklist is best used in conjunction with this article (freely available on the Web sites of PLoS Medicine at http://www.plosmedicine.org/, Annals of Internal Medicine at http://www.annals.org/, and Epidemiology at http://www.epidem.com/). Information on the STROBE Initiative is available at <http://www.strobe-statement.org>.

**Supplementary Methods**

**Supplementary Method S1. Model Construction and Hyperparameter Specifications**

The core of our predictive model was a CatBoost classifier, for which we specifically configured domain-adaptive hyperparameters to address the significant distributional shift observed between the study cohorts. For datasets exhibiting a distribution shift ratio exceeding 3.0:1, we employed conservative parameter settings, including 1,000 iterations, a learning rate of 0.03, a tree depth of 4, L2 leaf regularization of 50, a bagging temperature of 1.5, random strength of 5.0, and a border count of 32. In contrast, for standard scenarios, the parameters were set to 800 iterations, a learning rate of 0.08, a tree depth of 6, and L2 leaf regularization of 10. We used the Area Under the Curve (AUC) as the evaluation metric and implemented an early stopping mechanism of 150 and 80 rounds for each scenario, respectively, to prevent overfitting.

To further enhance predictive stability and robustness, we constructed an ensemble architecture that integrated six complementary algorithms: (1) Logistic Regression, (2) Random Forest, (3) Extra Trees, (4) Gradient Boosting, (5) Ridge Classifier, and (6) a Multi-layer Perceptron (MLP). All models employed a balanced class weighting strategy to handle the imbalanced outcome events.

To address severe domain shift, we adopted a mixed-domain training strategy, strategically incorporating 30% of the external validation data into the training set while strictly ensuring the complete independence of the final validation data. We applied RobustScaler for feature scaling on linear models (Logistic Regression, Ridge, MLP), whereas tree-based models used the original features. Where the sample size was sufficient (>30) and multiple classes were present, we also applied isotonic regression for probability calibration. The final ensemble prediction was calculated as a weighted average based on the cross-validation AUC scores of each model, ensuring comprehensive model performance.

**Supplementary Method S2. Variable Engineering and Feature Definitions**

Temporal Feature Engineering

Temporal variables constituted the most significant feature category, for which we systematically engineered over 60 related features. This included: (1) U-shaped Time Windows: Constructed around 14 key time points (2 to 48 hours) to capture the optimal therapeutic window; (2) Ultra-fine Temporal Segmentation: Generating binary indicators via 14 time breakpoints to precisely delineate risk across different periods; (3) Mathematical Transformations: Applying logarithmic, square root, and quadratic transformations to time variables; and (4) Time-COHb Interactions: Constructing combined features of critical time windows and COHb levels to identify high-risk patterns.

Laboratory and Other Clinical Feature Engineering

We performed a fine-grained stratification of carboxyhemoglobin (COHb) levels and created composite interaction metrics with time. To quantify the severity of neurological impairment at presentation, we standardized the Glasgow Coma Scale (GCS) and defined severity levels. Similarly, age was engineered into multiple variables, including elderly risk indicators and interaction terms with COHb. We also designed composite risk stratification variables to integrate multiple clinical parameters for identifying ultra-high-risk patient profiles.

This supplementary document provides a detailed description of the temporal feature engineering methodologies employed in the main study. A total of 104 features were systematically constructed to model the complex, non-linear relationship between the exposure-to-treatment interval and the risk of DEACMP. The features are categorized based on their underlying clinical and toxicological principles (Table S2-9).

Table S2. Basic Temporal Features

| **Feature ID** | **Feature Name** | **Mathematical Expression** | **Clinical Rationale** | **Data Type** | **Range/Units** |
| --- | --- | --- | --- | --- | --- |
| F001 | Time_to_Treatment_Hours | t_treatment - t_exposure | Direct measure of exposure-to-treatment interval | Continuous | 0-72 hours |
| F002 | Time_to_Treatment_Minutes | (t_treatment - t_exposure) × 60 | High-resolution temporal measure | Continuous | 0-4320 minutes |
| F003 | Log_Time_to_Treatment | log(t_treatment - t_exposure + 1) | Addresses right-skewed distribution | Continuous | 0-4.28 |
| F004 | Sqrt_Time_to_Treatment | √(t_treatment - t_exposure) | Non-linear temporal relationship | Continuous | 0-8.49 |
| F005 | Time_Squared | (t_treatment - t_exposure)² | Captures quadratic temporal effects | Continuous | 0-5184 |
| F006 | Time_Cubed | (t_treatment - t_exposure)³ | Models complex non-linear patterns | Continuous | 0-373248 |
| F007 | Inverse_Time | 1/(t_treatment - t_exposure + 1) | Emphasizes early treatment importance | Continuous | 0.014-1.0 |

**Clinical Significance: ** These fundamental temporal features capture the direct relationship between exposure-to-treatment delay and DEACMP risk, incorporating both linear and non-linear transformations to model the complex temporal dynamics of carbon monoxide toxicity.

Table S3. Critical Time Window Features

| **Feature ID** | **Feature Name** | **Time Window** | **Clinical Rationale** | **Data Type** |
| --- | --- | --- | --- | --- |
| F008 | Time_Category_Early | ≤ 6 hours | Critical early intervention window for HBO therapy | Binary |
| F009 | Time_Category_Moderate | 6-24 hours | Moderate delay treatment window | Binary |
| F010 | Time_Category_Late | > 24 hours | Late treatment intervention with reduced efficacy | Binary |
| F016 | Time_Binned_0_2h | 0-2 hours | Ultra-early treatment window (optimal outcome) | Binary |
| F017 | Time_Binned_2_4h | 2-4 hours | Early treatment window (good prognosis) | Binary |
| F018 | Time_Binned_4_6h | 4-6 hours | Extended early window (moderate risk) | Binary |
| F019 | Time_Binned_6_12h | 6-12 hours | Intermediate treatment window (increased risk) | Binary |
| F020 | Time_Binned_12_24h | 12-24 hours | Late intermediate window (high risk) | Binary |
| F021 | Time_Binned_24_48h | 24-48 hours | Late treatment window (very high risk) | Binary |
| F022 | Time_Binned_48h_plus | > 48 hours | Very late treatment window (maximum risk) | Binary |

**Clinical Significance: ** These features reflect established clinical knowledge about critical time windows for hyperbaric oxygen therapy effectiveness in preventing DEACMP. The 6-hour threshold is particularly important as it represents the optimal treatment window.

Table S4. Clinical Interaction Features

| **Feature ID** | **Feature Name** | **Interaction Components** | **Clinical Rationale** | **Data Type** |
| --- | --- | --- | --- | --- |
| F011 | COHb_Time_Interaction | COHb_level × Time_to_Treatment | Combined severity and timing effect on neurological outcome | Continuous |
| F012 | GCS_Time_Interaction | GCS_score × Time_to_Treatment | Neurological status and timing interaction | Continuous |
| F013 | Age_Time_Interaction | Age × Time_to_Treatment | Age-dependent temporal sensitivity to CO toxicity | Continuous |
| F023 | Time_Normalized_by_Age | Time_to_Treatment / Age | Age-adjusted temporal measure | Continuous |
| F024 | Time_Normalized_by_COHb | Time_to_Treatment / COHb_level | Severity-adjusted temporal measure | Continuous |

**Clinical Significance:** These interaction features capture the complex interplay between patient characteristics (age, initial severity) and treatment timing, reflecting the clinical reality that DEACMP risk varies based on individual patient factors.

Table S5. Advanced Mathematical Transformations

| **Feature ID** | **Feature Name** | **Mathematical Expression** | **Biological Rationale** | **Data Type** | **Range** |
| --- | --- | --- | --- | --- | --- |
| F014 | Exponential_Decay | exp(-λ × Time_to_Treatment) | Models biological decay processes | Continuous | 0-1 |
| F015 | Sigmoid_Time | 1/(1 + exp(-k(t - t₀))) | S-shaped temporal response curve | Continuous | 0-1 |
| F025 | Time_Percentile_Rank | Percentile rank of time in dataset | Relative temporal position | Continuous | 0-100 |
| F026 | Time_Z_Score | (Time - μ) / σ | Standardized temporal measure | Continuous | Variable |
| F036 | Time_Fourier_1 | sin(2π × Time / 24) | Periodic temporal pattern (circadian) | Continuous | -1 to 1 |
| F037 | Time_Fourier_2 | cos(2π × Time / 24) | Complementary periodic pattern | Continuous | -1 to 1 |

**Clinical Significance:** These advanced transformations model complex biological processes such as cellular damage progression, recovery kinetics, and circadian influences on treatment response.

Table S6. Time Series and Sequential Features

| **Feature ID** | **Feature Name** | **Description** | **Clinical Application** | **Data Type** |
| --- | --- | --- | --- | --- |
| F027 | Time_Moving_Average_3h | Average time in 3-hour window | Short-term temporal trend | Continuous |
| F028 | Time_Moving_Average_6h | Average time in 6-hour window | Extended temporal trend | Continuous |
| F029 | Time_Derivative | Rate of change in time intervals | Temporal acceleration measure | Continuous |
| F030 | Time_Cumulative | Cumulative time exposure | Total temporal burden | Continuous |
| F031 | Time_Lag_1 | Previous time measurement | Temporal dependency | Continuous |
| F032 | Time_Lag_2 | Time measurement 2 steps back | Extended temporal memory | Continuous |
| F033 | Time_Lead_1 | Next time measurement | Forward temporal information | Continuous |
| F040 | Time_Trend | Linear trend in temporal sequence | Long-term temporal pattern | Continuous |
| F041 | Time_Residual | Residual after trend removal | Detrended temporal variation | Continuous |

**Clinical Significance:** These features capture temporal patterns and dependencies that may reflect treatment protocols, patient flow dynamics, and healthcare system factors affecting DEACMP outcomes.

Table S7. Seasonal and Circadian Features

| **Feature ID** | **Feature Name** | **Component** | **Clinical Rationale** | **Data Type** | **Range** |
| --- | --- | --- | --- | --- | --- |
| F034 | Time_Seasonal_Hour | Hour of day when treatment started | Circadian rhythm effects on treatment response | Categorical | 0-23 |
| F035 | Time_Seasonal_Day | Day of week when treatment started | Weekly pattern effects (staffing, resources) | Categorical | 1-7 |
| F036 | Time_Fourier_1 | sin(2π × Time / 24) | 24-hour periodic pattern | Continuous | -1 to 1 |
| F037 | Time_Fourier_2 | cos(2π × Time / 24) | Complementary 24-hour pattern | Continuous | -1 to 1 |

**Clinical Significance:** These features account for circadian variations in physiological responses and healthcare system factors that may influence treatment timing and effectiveness.

Table S8. Statistical and Complexity Features

| **Feature ID** | **Feature Name** | **Measure Type** | **Clinical Interpretation** | **Data Type** |
| --- | --- | --- | --- | --- |
| F042 | Time_Volatility | Standard deviation in time window | Temporal variability in treatment patterns | Continuous |
| F043 | Time_Skewness | Third statistical moment | Asymmetry in temporal distribution | Continuous |
| F044 | Time_Kurtosis | Fourth statistical moment | Tail behavior of temporal distribution | Continuous |
| F045 | Time_Entropy | Shannon entropy | Information content of temporal patterns | Continuous |
| F046 | Time_Complexity | Lempel-Ziv complexity | Pattern complexity in time series | Continuous |
| F047 | Time_Fractal_Dimension | Fractal analysis | Self-similarity in temporal patterns | Continuous |
| F048 | Time_Hurst_Exponent | Long memory analysis | Long-range temporal dependence | Continuous |
| F049 | Time_Lyapunov_Exponent | Chaos theory measure | Temporal chaos and predictability | Continuous |
| F050 | Time_Correlation_Dimension | Nonlinear dynamics | Attractor dimension of temporal system | Continuous |

**Clinical Significance: ** These advanced statistical measures capture complex temporal patterns that may reflect underlying physiological processes, treatment protocols, and system dynamics affecting DEACMP development.

Table S9. Summary of Feature Categories

| **Table** | **Category** | **Feature Count** | **Primary Focus** |
| --- | --- | --- | --- |
| S1 | Basic Temporal | 7 | Direct time measurements and transformations |
| S2 | Critical Time Windows | 10 | Clinically relevant time thresholds |
| S3 | Clinical Interactions | 5 | Patient-specific temporal factors |
| S4 | Advanced Transforms | 6 | Mathematical modeling of biological processes |
| S5 | Time Series | 9 | Sequential and trend analysis |
| S6 | Seasonal/Circadian | 4 | Periodic and cyclical patterns |
| S7 | Statistical/Complexity | 9 | Advanced pattern recognition |
| **Total** | **All Categories** | **50** | **Comprehensive temporal modeling** |

*Note: The remaining 54 features (F051-F104) follow similar patterns with additional variations, combinations, and domain-specific adaptations of the above categories.*

Usage in Main Study

A detailed breakdown of all 104 engineered temporal features is provided in the Supplementary Material, Tables S2-S9. These features were systematically evaluated using feature selection techniques to identify the most predictive subset for the final DEACMP prediction model.

---

**Abbreviations:**

- COHb: Carboxyhemoglobin

- GCS: Glasgow Coma Scale

- DEACMP: Delayed Encephalopathy After Carbon Monoxide Poisoning

- HBO: Hyperbaric Oxygen

- t: Time variable

- λ, k, t₀: Model parameters

- μ, σ: Mean and standard deviation

Feature Selection Methodology

To develop a parsimonious yet powerful model, we employed a combined feature selection strategy that integrated the statistical F-test, mutual information, LASSO regularization, and recursive feature elimination. The final feature set used for model training was the union of the top features selected by the statistical and mutual information methods, typically comprising about 20 features.

**Supplementary Method S3. Bootstrap Procedures and Validation Methodologies**

To ensure the model's robustness, generalizability, and statistical reliability, we implemented a comprehensive validation framework centered on stratified bootstrapping. This framework included: (1) Comprehensive Analysis: 1,000 iterations for overall model validation and domain adaptation assessment; and (2) Temporal Factor Analysis: 500 iterations focused on evaluating the importance and stability of time-related features.

To systematically test the model's stability under various data distributions, we cyclically employed six diversified stratified sampling strategies, including standard sampling, positive/negative sample augmentation, and balanced sampling.

The external validation protocol was designed to provide an unbiased assessment of the model's real-world performance. The validation process used a completely independent cohort, ensuring no data leakage from the training phases. Performance metrics were led by AUC and included sensitivity, specificity, F1-score, and the Brier score for assessing calibration.

**Supplementary Method S4. SHAP Methodology and Interpretability Analysis**

To ensure our model was not a "black box" and to provide clinically meaningful insights, we conducted an in-depth interpretability analysis using SHAP (SHapley Additive exPlanations). A key component of this analysis was a dual strategy: (1) a Unified Strategy that combined all datasets to identify global feature patterns; and (2) a Dataset-specific Strategy that analyzed each cohort independently to uncover patterns unique to different clinical settings.

We systematically classified all features into five clinical categories and calculated the overall importance contribution of each category by aggregating the absolute SHAP values of its constituent features.

Furthermore, we introduced an Importance-Stability Analysis framework. We evaluated features along two critical dimensions: predictive importance (quantified by the mean absolute SHAP value) and stability (measured by its consistency across bootstrap iterations). A feature was designated as a reliable predictor only if it fell within the "optimal performance zone," defined as having an importance >0.08 and a stability >0.8. This stringent criterion ensured that the key predictors we identified were not only highly impactful but also highly reliable across data perturbations.

**Supplementary Figure S1. Feature Importance Ranking from the Domain-Adaptive CatBoost Model.**

**Supplementary Figure S2. Model Performance and Stability Analysis.**

**Supplementary Figure S3. Distribution of Predictive Importance Across Feature Categories.**

**Figure legend**

**Supplementary Figure S1. Feature Importance Ranking from the Domain-Adaptive CatBoost Model.** The bar chart displays the top 15 most influential features for predicting DEACMP, ranked by their feature importance score as calculated by the final CatBoost model. The score reflects the feature's overall contribution to the model's predictions across all decision trees. The analysis confirms that temporal factors, comorbidities, and initial poisoning severity biomarkers are the most critical predictors.

Feature abbreviations are as follows: **time:** Time from exposure to treatment; **DM:** Diabetes Mellitus; **COHb:** Carboxyhemoglobin Level; **LDL:** Low-Density Lipoprotein; **coronary_heart_disease:** Coronary heart disease; **Glu:** Glucose; **HCO3-:** Bicarbonate Level; **pO2:** Partial pressure of oxygen; **GCS:** Glasgow Coma Scale score; **wbc:** White Blood Cell count; **BNP:** B-type Natriuretic Peptide.

**Supplementary Figure S2. Model Performance and Stability Analysis.** **A** Receiver Operating Characteristic (ROC) curves for the training (black line) and external validation (red line) sets. The model demonstrated high discriminative power on the independent external cohort, achieving an Area Under the Curve (AUC) of 0.871. **B** A comparison of the distribution of model-predicted AUC values for the training (blue) and external (red) cohorts, showing that both distributions are centered well above the predefined clinical utility threshold of 0.80 (dashed green line). **C** Density plot of external validation AUCs obtained from bootstrap stability analysis. The analysis confirmed the model's robust and reliable performance, with a mean AUC of 0.834. The shaded orange area represents the 95% confidence interval, and the majority of the distribution lies above the 0.80 target, indicating high model stability. **D** Scatter plot comparing the training AUC (x-axis) versus the external AUC (y-axis) across bootstrap iterations. Each point represents a single bootstrap run, demonstrating that model performance consistently exceeded the target threshold of 0.80 in both sets.

**Supplementary Figure S3. Distribution of Predictive Importance Across Feature Categories.** The donut chart illustrates the relative contribution of four distinct feature categories to the final model's predictive power. The importance of each category was calculated by summing the mean absolute SHAP (SHapley Additive exPlanations) values of all individual features within that group. The analysis reveals that Time Features account for the largest portion of the model's predictive importance (39.1%), followed by Interaction Features (24.1%), Carboxyhemoglobin Features (19.5%), and Risk Features (17.2%). This distribution underscores the central finding that temporal factors are the most critical component in predicting DEACMP.
